# Design and Implementation of a Smart Sensor for Respiratory Rate Monitoring

**DOI:** 10.3390/s140203019

**Published:** 2014-02-14

**Authors:** Juan Aponte Luis, Laura M. Roa Romero, Juan Antonio Gómez-Galán, David Naranjo Hernández, Miguel Ángel Estudillo-Valderrama, Gerardo Barbarov-Rostán, Carlos Rubia-Marcos

**Affiliations:** 1 OnTech Security Enterprise, C/Lucena del Puerto, Huelva E-21002, Spain; E-Mail: juan.aponte@ontech.es; 2 Biomedical Engineering Group, University of Seville, Avda de los Descubrimientos, s/n, Seville E-41092, Spain; E-Mails: laura@esi.us.es (L.M.R.R.); davidazuaga@gmail.com (D.N.H.); m.estudillo@gmail.com (M.Á.E.-V.); gbarbarov@gmail.com (G.B.-R.); 3 Department of Electronic Engineering, Computers, and Automatic, University of Huelva, Ctra Huelva, La Rábida, s/n, Huelva 21819, Spain; E-Mail: carlmass@diesia.uhu.es

**Keywords:** capacitive technology, respiratory rate monitoring, smart sensor

## Abstract

This work presents the design, development and implementation of a smart sensor to monitor the respiratory rate. This sensor is aimed at overcoming the drawbacks of other systems currently available in market, namely, devices that are costly, uncomfortable, difficult-to-install, provide low detection sensitivity, and little-to-null patient-to-patient calibration. The device is based on capacitive sensing by means of an LC oscillator. Experimental results show that the sensor meets the necessary requirements, making feasible the proposed monitoring system with the technology used.

## 1. Introduction

Respiratory rate monitoring plays an important role in the control and follow-up of highly prevalent diseases, such as COPD [1] or sleep apnea [2,3]. With this aim, respiratory rate detection becomes a frequently-used alternative, also used with other physiological variables, such as, pulse oximetry and heart rate [4,5].

Next, analysis of the main detection techniques—particularly those used in apnea—are completed, but its conclusions can be extended to the diagnosis of other respiratory pathologies. Polysomnography is the standard diagnosis technique for apnea [6,7]. However, it involves a long and laborious procedure demanding the presence of qualified healthcare staff, it is costly and also unpleasant for patients. Respiratory polygraphy is another alternative technique, which involves reduced complexity, low waiting time and little monetary cost, but it is an invasive technique.

The fact that they are obstructive systems from the patient's viewpoint, together with the complexity involved by their use, has led to the research into new monitoring systems, such as the so-called smart beds by means of pressure sensors [8]. However, due to the nature of this method, respiratory measurements can be affected by the patient's weight and position. On the other hand, the electronic nose and the respiratory polygraph are aimed at simplifying diagnosis, as they are between three and four times cheaper than traditional technology [9]. Another technique currently under development for early diagnosis is the study of the voice, both in apnea detection and evaluation of the patient's risk to develop it. Systems based on piezoelectric sensors integrated into a belt have also been used [10].

However, in spite of all efforts, current systems still involve a series of drawbacks that hinder early detection of sleep apnea. The main drawbacks detected so far could be summarized as follows: (1) they are invasive and annoying for patients; (2) their long duration; (3) they are based on algorithms with scarce patient-to-patient calibration; (4) they are costly and (5) complex to use.

This work presents the development of a low-cost and non-obstructive sensor to monitor respiratory rate for sleep apnea follow-up and diagnosis purposes. The paper is organized as follows: Section 2 contributes a preliminary study on the technological feasibility of capacitive sensing. Section 3 describes each of the stages of the proposed sensing system, as well as the necessary materials and methods. Section 4 presents the simulation and experimental results obtained in terms of sensitivity, interferences and respiratory rate detection. Finally, conclusions are drawn in Section 5.

## 2. Preliminary Considerations

The importance of using reliable, affordable and highly performing sensors for noninvasive medical therapies is progressively growing, since trends forecast a significant increase in patient follow-up and control from home [3]. The technology based on capacitive sensing was chosen for respiratory rate measurement instead of other solutions (see [11–13]) because it involves the following advantages:

– The sensor's price would be low, since it is made of standard electronic components;
– Capacitive sensors are widely used in industry and prove rather efficient. Therefore, we consider that adapting them to a different sector (namely, healthcare) could be achievable and beneficial;
– Their inner configuration allows them to meet the requisite of avoiding contact between the electrodes and the patient;
– The resolution of capacitive sensors in short distances is rather high;
– The most relevant parameters for alterations to take place in the operating frequency of capacitive sensors are dielectric variations produced between the electrodes. In our case, the critical dielectric is air inside the lungs.

One of the applications equipped with capacitive sensors has been successfully applied to monitor intraocular pressure [14,15] and intracranial pressure [16]. Pressure measurements are the activities in which capacitive sensors have been most extensively used in medicine, although these capacitive systems have also been applied in some other relevant projects in the medical field. Accelerometers are an example of the foregoing, as they are instruments used to measure inclination in body segments and daily physical activity in rehabilitation patients [17]. The use of capacitance in these devices to measure displacement leads to significantly improved sensitivity. Another use of capacitive sensors includes the diagnosis of pulmonary diseases through humidity measurements (humidity sensors). In this kind of devices, a chemically absorbent layer, commonly a polymer, is placed between the parallel electrodes in a capacitor. Thus, humidity is detected as a change in capacitance due to the variation in the dielectric constant when the water molecules in the polymer are absorbed [11]. On the other hand, capacitive sensors have also been used to monitor respiratory rate in real time [13]. In this case, an abdominal belt was designed and fabricated for efficient respiratory rate measurement by means of a differential capacitive circuit with screening.

Completed studies prove that the use of capacitive sensors in medical applications is progressively increasing due to their advantages: reduced size, high sensitivity, low cost, and reduced power consumption. Among capacitive sensors, oscillator-based capacitive sensors are a widely extended technology. This kind of sensors generates a sinusoidal signal whose frequency is set by the value of the inductor and the capacitor used. Oscillation frequency is used as a parameter to determine the value of the capacitance to be measured. The main advantages involved by oscillator-based capacitive sensors are the following:

– High sensitivity in frequency relative to variations in the capacitance to be measured;
– Frequency stability in case of different phenomena such as vibrations, temperature changes, supply voltage changes, *etc*.

This kind of capacitive sensors is formed by an oscillator and the measured capacitance, which comprises the capacitance of the electrodes and the dielectric capacitor. In our case, we will deal with two dielectrics: the air and the human body (skin, liquids, *etc.*), which will modify the capacitor's value. One of the most relevant features for the sensitivity of the designed sensor consists on obtaining considerable variations in the operating frequency through small changes in the capacitance of the electrodes. This particularity is provided by the oscillators by means of its resonant network. The oscillator is the key element in this kind of capacitive sensors, whose correct operation will be essential for the efficacy and sensitivity of the sensor itself.

## 3. Description of the Proposed System

**Figure 1.**
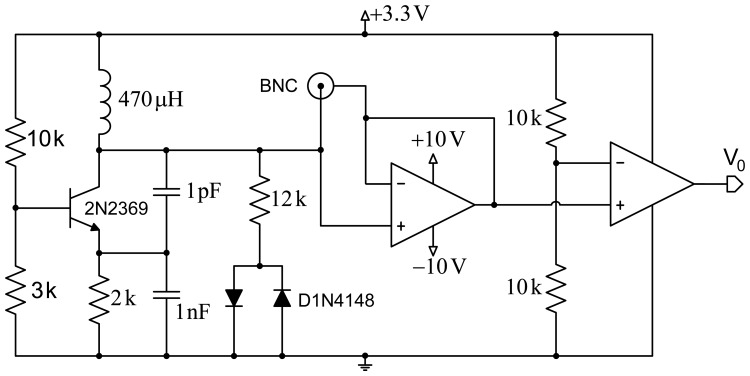
Scheme of the sensing system based on a Colpitts oscillator, together with conditioning stages.

Figure 1 shows a scheme of the proposed design. The first stage comprises a Colpitts oscillator designed to achieve a fairly high quality factor (Q). Good frequency stability is intended, and demands optimizing the transistor's point of operation, as well as using a transistor with a very low collector-base junction capacitance. The second design stage includes a network formed by a resistor and two ground-derived antiparallel diodes that are coupled to the oscillator for improved performance. This contributes several improvements to the system: avoiding oscillation in the sensor circuit based on the operational amplifier, improved quality factor (Q), and setting a constant input voltage to the operational amplifier for increased system stability.

The third stage allows reducing the effect of the parasitic capacitances generated by the circuit itself, by the electrodes or the human body through the creation of a shield with an operational amplifier configured as a voltage follower. Finally, a signal conditioning stage formed by an additional operational amplifier, working in this case as a comparator, provides a square wave with constant voltage. This signal is applied to a microcontroller to process it and obtain the oscillator's operating frequency.

The key to success in the design of an oscillator-based capacitive sensing system that meets the expectations of obtaining good enough resolution in the measured frequency for capturing the respiratory rate, is to produce an optimized oscillator design and choosing appropriately in the design of the electrodes and their size. In any case, it is equally important to study in detail all the parasitic capacitances involved in the measurement of sensor and all external interferences that may change the frequency measurement.

### 3.1. Optimized Oscillator Design

LC oscillators are circuits formed by a coil and a capacitor in parallel (LC tank). Its operation is based on the exchange of electric and magnetic energy between the capacitor and the coil, respectively. These oscillators have been widely applied in industrial fields to detect the presence of objects and measure levels in deposits and containers, since it is low-cost, highly sensitive, and requires no direct contact with the measured object.

This kind of oscillator can generate a sinusoidal wave by means of amplification and feedback. Its active element is usually one only transistor (bipolar transistor or FET) or an operational amplifier. The common base configuration of the Colpitts oscillator was used in the design of the proposed sensor device, as it is widely used in industrial applications due to its robustness. However, its use in biomedical applications has been somewhat more reduced. Oum *et al.* [18] used a Colpitts-based scheme for respiratory rate measurement and obtained a simple, low-cost and highly sensitive design. The present work proposes several improvements that provide more sensitive respiratory rate measurements under more adverse conditions. Our main aim is designing and implementing an oscillator circuit that minimizes the parasitic capacitances generated by the circuit itself and allows realistic measurement of the capacitance produced between the electrodes and the human body with high sensitivity to changes of capacitance.

The frequency stability is the capacity of an oscillator to keep a fixed frequency, and is often considered either short- or long-term. The former is mainly affected by fluctuations in DC operating voltages, while the latter is a function of component age, temperature changes and environmental humidity. In the aforementioned LC tank circuit oscillators, quality factors Q are relatively low, thus allowing the resonant tank circuit to oscillate over a wide range of frequencies. Frequency stability is provided as a frequency-change percentage (tolerance) relative to the desired value.

**Figure 2.**
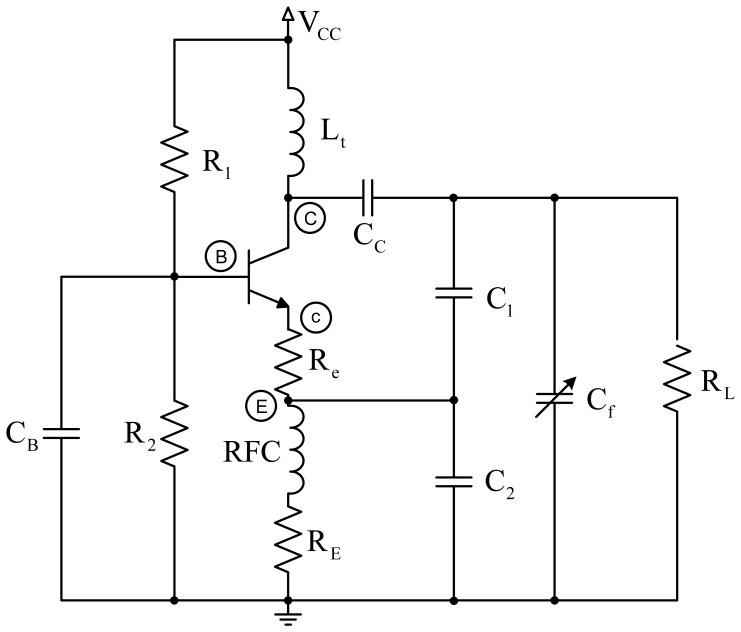
General scheme of the Colpitts oscillator.

Figure 2 shows the scheme of the Colpitts oscillator in common-base configuration used for the proposed design. The tank circuit comprises self-induction *L_t_* and capacitances *C*_1_ and *C*_2_. Capacitance *C_f_* is the capacitance produced by the electrodes placed on the human body. *R_e_* is a small resistor included to avoid the oscillator performance depends on the transistor's input impedance. Load resistor is *R_L_*, which is connected to the collector through decoupling capacitor *C_C_*. Resistors *R*_1_, *R*_2_ and *R_E_* are used for circuit polarization.

The oscillator design is usually conditioned by specifications (usually regarding the oscillation frequency and the power to be supplied to a specific load resistance), and the available components. However, in our case, the oscillator design is determined by the proposed capacitance *C_f_*, the quality factor Q that we intend to assure, and the maximum power supplied to the load. Capacitance *C_f_*, formed by both electrodes, which would be connected at the patient's chest and back in a first approximation, is very similar to the capacitance produced by the human body. A capacitance of 150 pF was taken as a reference according to previous studies [19–21].

For the analysis of the power supplied to the load, the oscillator should be designed for conditions of maximum transfer to the load, which means that *R*_0_ must equal half *R_L_*, where *R*_0_ is the equivalent resistance of the circuit with no load *R_L_*. The oscillation frequency directly depends on the coil and the capacitance, as shown by the equation: $f_{0}=1/\left(2\pi \sqrt{L_{t}C_{t}}\right)$, where *C_t_* = [*C*_1_*C*_2_/(*C*_1_ + *C*_2_)] + *C_cb_*, and *C_cb_* is the parasitic capacitance between the collector and the transistor base. The quality factor is expressed by *Q* = 2π*f*_0_*R*_0_*C_t_*. This is an important parameter, since the higher it is, the lower the noise of the oscillator will be.

The values of the components in the circuit are determined to achieve permanent oscillation conditions in the system. A coil with a higher value than the one in [18] was used to improve the circuit sensitivity, although an increased external noise is also expectable. An RF-type transistor with small collector-base capacitance (around 0.5 pF) was used to reduce the impact of the circuit input voltage on the circuit sensitivity. The values of the base resistance of the transistor were chosen from the optimal point on the load line, and thus achieve the transistor operates in optimum oscillation conditions.

### 3.2. Reduction of the Effect of Parasitic Capacitances

**Figure 3.**
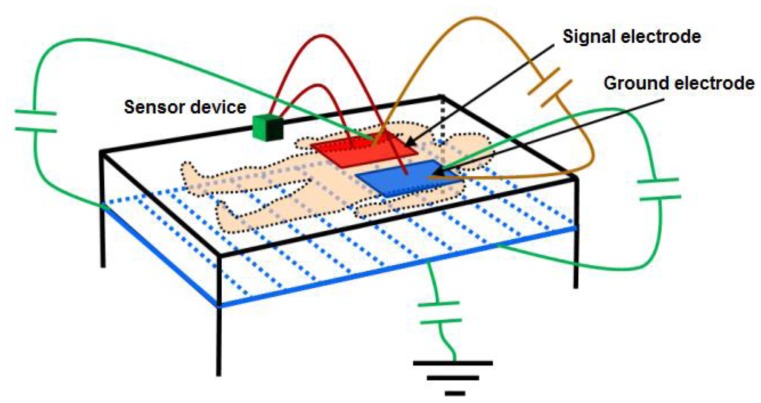
Proposed monitoring scheme.

With the objective of reducing the parasitic capacitances generated between the electronic circuit itself and the electrodes, relative to the capacitance produced between the electrodes and the human body, setting a screen between the electrodes and the ground has been considered. This screen is obtained by setting the metallic structure of the bed at the same potential as the oscillator output through an operational amplifier in the configuration of the voltage follower. Figure 3 shows the proposed scheme and the most important capacitances affecting measurement.

The voltage follower configuration of the operational amplifier provides the advantages of very high input impedance and small output impedance. Using an operational amplifier that keeps these features in working frequencies, the capacitance created between the screen and the ground electrode will be very small, since the operational amplifier's output impedance is small. Although the capacitance existing between the signal electrode and the screen may physically have a high value (≈150 pF), it can be considered negligible since the values of the voltage potential are practically the same. Finally, the screen minimizes the effects of the capacitance existing between the ground electrode and the earth, since this configuration favors that field lines from the signal electrode avoid the earth in favor of a more direct path towards the ground electrode. Other parasitic effects are eliminated by setting this voltage potential also in the copper mesh surrounding the coaxial cables that connect the electrodes to the device.

### 3.3. Electrode Design

In this design stage, the different configurations used in the study of the effect of electrodes are described: electrode size, geometrical shape, material, position and number of electrodes. The scheme proposed to monitor respiratory rate considers placing a pair of metallic electrodes covered by an insulating material on a bed. The body of the monitored patient is placed on the electrodes so that the capacitance generated between them is affected by the human body. With the aim of obtaining appropriate setting, a series of co-planar electrode configurations were tested: (1) two 9 × 24 cm rectangular electrodes separated by 2 cm; (2) two electrodes formed in 8 arrays of 55 × 1 cm alternated in an interdigitated array with 1 cm separation; (3) two 14 cm × 25 cm rectangular electrodes in different arrays separated by 1, 2 and 3 cm; and (4) two 22 cm × 4 cm rectangular electrodes with 1, 2 and 3 cm separations. A scheme composed only by the signal electrode (10 × 10 cm) was also tested; in this case, capacitance was only formed with the ground plane of the device.

### 3.4. Output Signal Conditioning

Finally, the last stage simplifies the signal transduction method used in [18] (derivative, amplification and envelope detection). In this stage an operational amplifier set as a comparator transforms the input sine wave into an amplitude-limited square wave. A microcontroller (PIC18LF2431 by Microchip Corp., Chandler, AZ, USA) counts the number of the rising edges during a given time period and, according to this data, sets the instantaneous oscillation frequency of the capacitive sensor. The system also includes a ZigBee module to send data remotely via wireless communication.

## 4. Simulation and Experimental Results

The proposed sensor device was simulated and fabricated to test its efficiency, robustness and suitability for respiratory rate measurement. With this purpose, sensitivity and interference with other receptors were analyzed first. Subsequently, the fabricated device was tested on two patients with different physical features.

### 4.1. Study of Sensitivity

The sensitivity of the proposed capacitive sensor is a critical parameter for reliable respiratory rate detection, since the detection system is based on the measurement of the capacitance variations that usually take place as a consequence of these two factors:

– Changes in the dielectric generated between the electrodes of the capacitive sensor;
– Increased parasitic capacitances produced between the electrodes of the capacitive sensor and both the floating and fixed ground.

A sensitivity study was accomplished to determine the appropriate capacitance ranges for robust device operation. For this analysis, the developed design was simulated by means of software package LT Spice IV. With this purpose, the capacitance with the human body (*C*_MEASURED_) was varied, as it depends on electrode type, shape and size, as well as on their placement regarding the human body.

**Figure 4.**
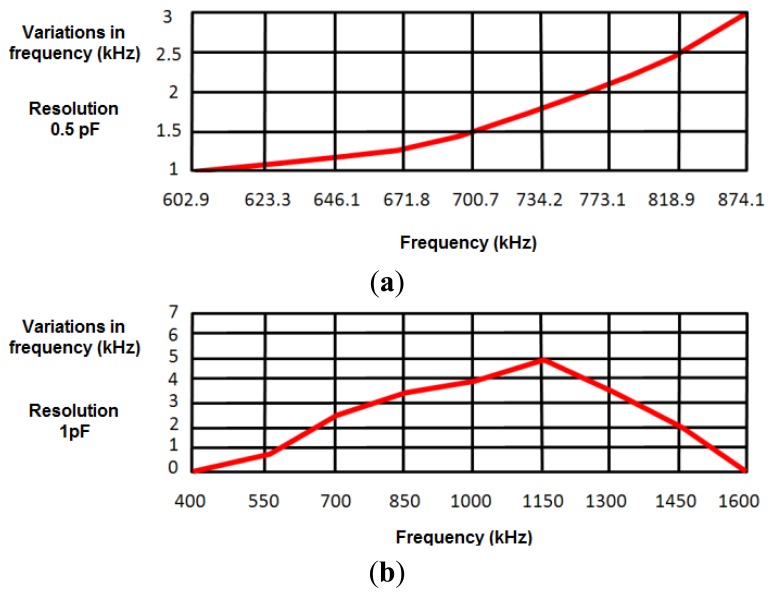
Frequency variations generated by a 0.5 pF (**a**) and 1 pF (**b**) increase in C_MEASURED_.

To develop a robust system that minimizes the effects of external interferences and noise, a safety margin of 1 kHz was set in measurements (experimentally, the error margin was set around ±200 Hz). A first experimental study provided a value around 50 pF for the capacitance with the human body, oscillating the sensor at 874.2 kHz. Taking this value as a reference in simulations, the capacitance value was increased 0.1 pF to test the sensitivity of the design to capacitance variations in the human body. This increase in the capacitance value reduced the oscillation frequency around 1 kHz, just in the limit of the safety margin. Therefore, the device cannot be considered inappropriate to detect 0.1 pF capacitance variations. Figure 4a shows frequency variations produced by a 0.5 pF increase in the human body capacitance value, the latter being in turn related to the oscillation frequency. Results show that the sensor device is sensitive to 0.5 pF variations in the range of frequencies from 602.9 kHz (130 pF) to 874.10 kHz (50 pF).

Figure 4b shows the frequency variations produced by a 1 pF increase in human body capacitance. Therefore, the proposed sensor can be considered sensitive to 1 pF variations in the range of frequencies between 508 kHz (50 pF) and 1,488 kHz (200 pF). As shown in Section 4.3, both ranges of sensitivity prove enough to monitor respiratory movements.

### 4.2. Study of Interferences

**Figure 5.**
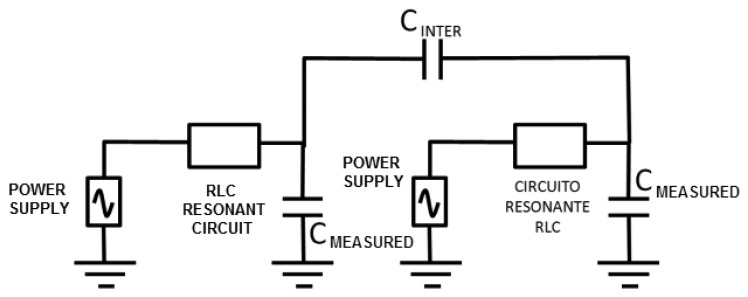
Scheme used in the simulation of interferences between sensor devices.

One of the important factors that can affect the sensor is the interferences from other neighboring devices. This effect was modeled through a capacitance between both circuits (C_INTER_, see Figure 5). The LTSpice IV software allows determining a first approach of the interference-free minimum distance.

Negligible interference was obtained for capacitance values in both 30 and 50 pF circuits (two capacitances associated to experimental oscillation frequencies) and a capacitance value of 0.1 pF for C_INTER_. The C_INTER_ value corresponds to an approximate distance of 2 m, which was experimentally set by means of a capacitance meter.

**Figure 6.**
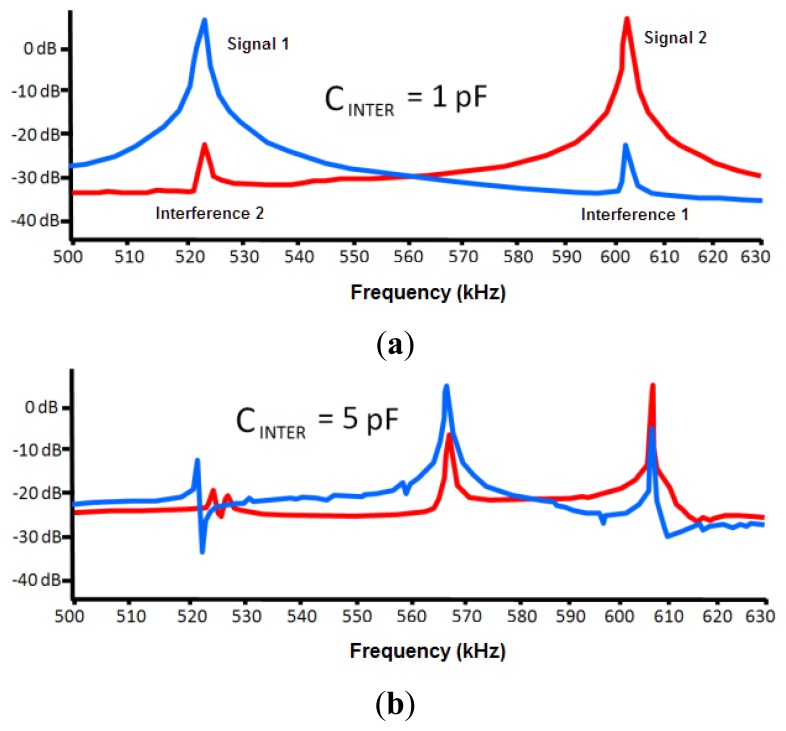
Simulation of the frequency signals for two sensor devices in two cases of the study of interferences: (**a**) C_INTER_ = 1 pF; (**b**) C_INTER_ = 5 pF.

If a 1 pF value is set to C_INTER_, the interference remains negligible, as shown in Figure 6. A capacitance meter experimentally related this capacitance to an approximate distance of 1 m. However, a high interference is shown for a value of 5 pF. This value is experimentally associated to a distance of 30 cm, which was set as a first approach to the safety limit among devices. A high interference originates for a value of 10 pF that is associated to a distance of 10 cm.

### 4.3. Respiratory Rate Detection

**Figure 7.**
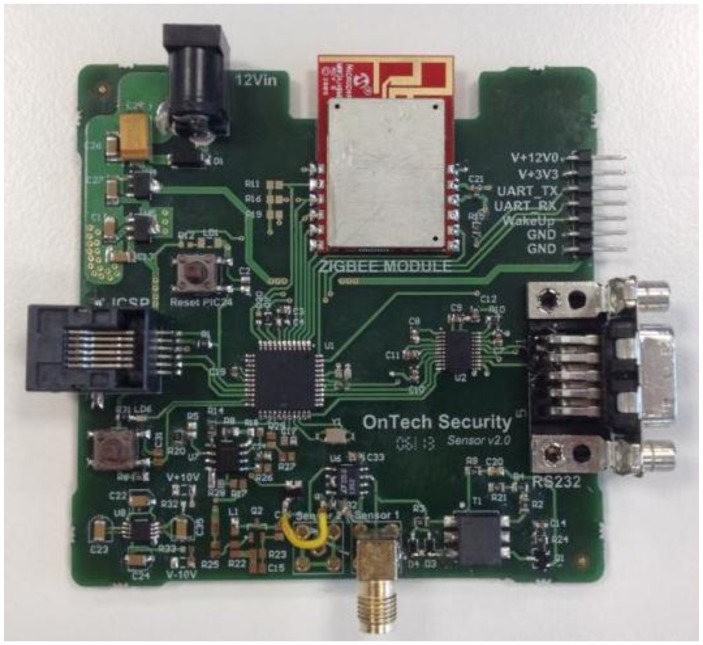
Photograph of the proposed detection system, composed by the capacitive sensor and the conditioning electronics.

To test the reliability of the proposed sensor, an experimental test was organized according to the designed system, as shown in Figure 7.

**Figure 8.**
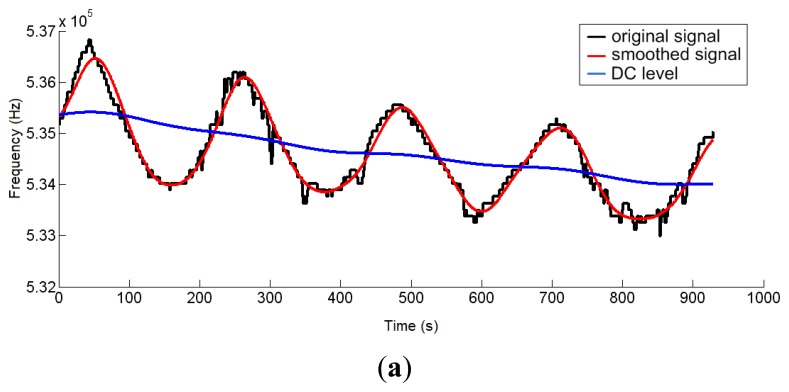

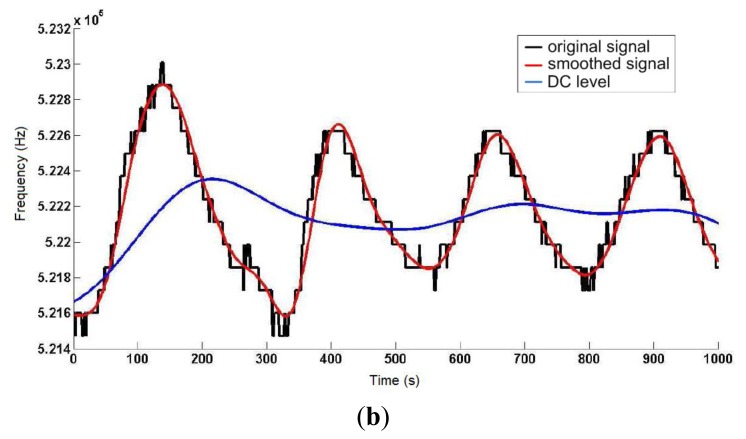
Resulting respiratory signal after second-stage processing of both volunteer patients. (**a**) Patient 1; (**b**) Patient 2.

On a 2 m × 2 m inflatable mattress located 30 cm over the ground, two electrodes were placed according to the arrangements described above. In this first study, two males aged 35 and 29, 1.82 and 1.75 m tall, and weighing 99.5 and 80 kg, respectively, were asked to lie in different positions (face up, face down, on one side) on the electrodes, placed at the level of their chest. The value of the instantaneous oscillation frequencies of the sensor device were sent to a computer through the serial port at rate of 32 samples per second. These data were processed by Matlab software by means of a 3-stage algorithm. In the first stage, a fourth-order Butterworth low pass filtering was accomplished with a cutoff frequency of 0.32 Hz to smooth the signal and eliminate noise components (see Figure 8). In the second stage, a fourth-order Butterworth low pass filtering, but in this case at a much lower cutoff frequency (0.064 Hz), allowed extracting the DC component of the signal, since a drift in the DC level was experimentally detected.

**Figure 9.**
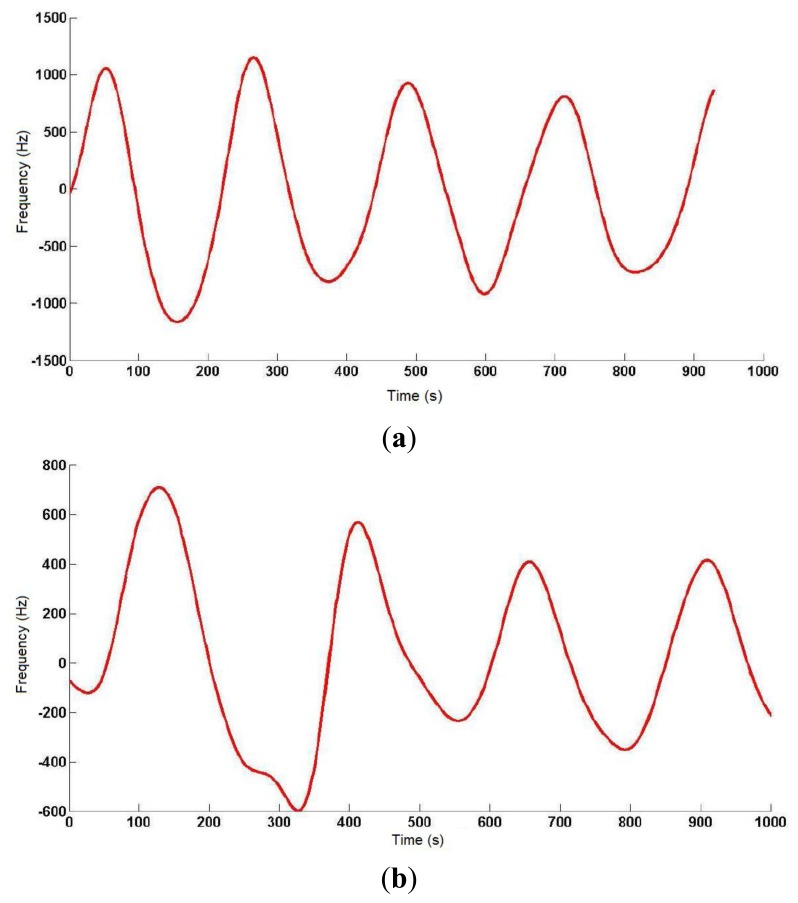
Output signal after the third-stage processing of both volunteer patients. (**a**) Patient 1; (**b**) Patient 2.

Figure 9 shows the output signal of the third stage, which is the difference between the signals from the previous two stages. The resulting signal reflects the respiratory movements more clearly, and the instantaneous respiratory frequency can be obtained by dividing the number of crossovers of the signal (only in the rising or falling edges) by the period of sample processing.

The respiratory signal could be obtained in all cases, although the oscillator's parameters had to be adjusted to adapt it to the user's specific features (patient-to-patient calibration), since the generated capacitance differed in each of them. The identification process was completed visually with the collaboration of the monitored individual, as a direct correspondence was observed between thoracic movement and transitions in the obtained signal curve. The best results were obtained for the interdigitated electrodes with the patient face up, and for only one signal electrode (10 cm × 10 cm) with the patient face down.

## 5. Discussion and Conclusions

The importance of follow-up in respiratory diseases can be observed in the progressively increasing efforts to develop new respiratory rate detection methods that help overcome the drawbacks shown by present-day available alternatives. In this context, this work presents a new approach to this problem based on new trends of smart monitoring. With this aim, the system makes use of noninvasive, low-cost and highly sensitive detection technologies according to the capacitive sensing principles of LC oscillators. A detailed study of the main interferences occurring in the proposed sensor was accomplished, as well as a deep analysis of sensitivity regarding the capacitance to be measured.

Simulation results show a wide operating range of the proposed capacitive sensor. Therefore, the respiratory rate can be measured in any patient with no sensor modification. We observed that the interferences from devices working at similar frequencies nearby may lead to measurement errors. To reduce external interferences, the development of a screening system is proposed, as it was observed to reduce the existing parasitic capacitances.

The results obtained in this research suggest that this approach meets the starting requirements and makes the presented system feasible to monitor respiratory diseases. The presented sensor can be integrated into a multimodal platform for online healthcare and remote development of the patient-to-patient calibration of the detection algorithm. The developed sensor device can be extended to other related applications with heart rate measurements or ECG by means of capacitive sensors or to detect falls in patients or the elderly.
